# Structural Analysis of the Rubisco-Assembly Chaperone RbcX-II from *Chlamydomonas reinhardtii*

**DOI:** 10.1371/journal.pone.0135448

**Published:** 2015-08-25

**Authors:** Andreas Bracher, Thomas Hauser, Cuimin Liu, F. Ulrich Hartl, Manajit Hayer-Hartl

**Affiliations:** Department of Cellular Biochemistry, Max-Planck-Institute of Biochemistry, Martinsried, Germany; Tsinghua University, CHINA

## Abstract

The most prevalent form of the Rubisco enzyme is a complex of eight catalytic large subunits (RbcL) and eight regulatory small subunits (RbcS). Rubisco biogenesis depends on the assistance by specific molecular chaperones. The assembly chaperone RbcX stabilizes the RbcL subunits after folding by chaperonin and mediates their assembly to the RbcL_8_ core complex, from which RbcX is displaced by RbcS to form active holoenzyme. Two isoforms of RbcX are found in eukaryotes, RbcX-I, which is more closely related to cyanobacterial RbcX, and the more distant RbcX-II. The green algae *Chlamydomonas reinhardtii* contains only RbcX-II isoforms, CrRbcX-IIa and CrRbcX-IIb. Here we solved the crystal structure of CrRbcX-IIa and show that it forms an arc-shaped dimer with a central hydrophobic cleft for binding the C-terminal sequence of RbcL. Like other RbcX proteins, CrRbcX-IIa supports the assembly of cyanobacterial Rubisco in vitro, albeit with reduced activity relative to cyanobacterial RbcX-I. Structural analysis of a fusion protein of CrRbcX-IIa and the C-terminal peptide of RbcL suggests that the peptide binding mode of RbcX-II may differ from that of cyanobacterial RbcX. RbcX homologs appear to have adapted to their cognate Rubisco clients as a result of co-evolution.

## Introduction

Life on earth depends on fixation of atmospheric CO_2_ into organic compounds by bacteria, algae and plants. The key enzyme for this process ribulose-1,5-bisphosphate carboxylase/oxygenase (Rubisco) catalyzes the carboxylation of the five-carbon sugar ribulose-1,5-bisphosphate (RuBP) which is converted into two molecules of 3-phosphoglycerate. The other enzymes of the Calvin–Benson–Bassham cycle subsequently use reduction equivalents and ATP produced in the light reaction of photosynthesis to regenerate RuBP and produce triose phosphate to fuel anabolic pathways. The most prevalent form of Rubisco (form I) consists of a complex of eight catalytic large subunits (RbcL), forming a *D*4-symmetric core, and eight regulatory small subunits (RbcS), capping the RbcL_8_ complex at both ends [1]. RbcL sequences exhibit remarkable conservation across phyla. Nevertheless, based on sequence diversity of the RbcL subunits, four subgroups of form I Rubisco, IA-ID, can be distinguished [2, 3]. The economically most important form IB is found in so-called green organisms: cyanobacteria, green algae and plants.

While there is a plethora of data on Rubisco structure, function and catalysis [1, 4], the pathways of subunit folding and oligomeric assembly are only beginning to emerge [5]. In green algae and plants, the RbcL subunits are chloroplast encoded and synthesized in the chloroplast stroma, the site of carbon fixation. In contrast, the RbcS subunits are nuclear-encoded, translated in the cytosol and imported into chloroplasts [6]. Newly-synthesized RbcL subunits associate with the chloroplast chaperonin Cpn60 [7], the homolog of bacterial GroEL, initially suggesting that the chaperonin mediates Rubisco assembly [8].

Recent reconstitution of cyanobacterial form I Rubisco in vitro demonstrated that the chaperonin mediates RbcL folding, while assembly of the RbcL_8_ core complex requires the additional factor RbcX [9, 10]. Co-expression of RbcX was also required for the recombinant production of the Rubisco from the cyanobacterial species *Synechococcus* sp. PCC7002 (Syn7002) and increased the efficiency of functional expression of *Synechococcus elongatus* PCC6301 (Syn6301) Rubisco [11, 12]. In most cyanobacteria, the gene for RbcX is located between the *rbcL* and *rbcS* genes within a single operon [13]. Mutation or deletion of *rbcX* was found to reduce the level of functional Rubisco in PCC7002 and *Synechococcus elongatus* PCC7942 [11, 14]. RbcX is highly conserved in all prokaryotes and eukaryotes containing form 1B Rubisco [15]. Structural analysis showed that RbcX is a dimeric, α-helical protein of ∼15 kDa subunits [12, 16–18]. The dimer structure has a central hydrophobic cleft which serves as binding site for the C-terminal sequence motif EIKFEF(E/D) in RbcL sequences [12, 15]. The peptide binds in an extended conformation with the Phe sidechains reaching into hydrophobic pockets [10, 12]. In addition, the boomerang-shaped RbcX dimer has conserved residues at the corners that mediate interactions with the adjacent RbcL subunit [10, 12]. Thus, RbcX binding clamps the RbcL anti-parallel dimer together and facilitates the assembly of the RbcL_8_ core complex. The RbcL-RbcX interaction is dynamic, allowing the displacement of RbcX from RbcL_8_ complexes by RbcS to form the holoenzyme. RbcX therefore functions as a Rubisco assembly chaperone.

Many eukaryotes have two RbcX homologs, one that closely resembles the cyanobacterial ortholog, RbcX-I, and a more distantly related homolog, RbcX-II [12]. The RbcX-I and RbcX-II from *Arabidopsis thaliana* have been characterized and crystallized, named AtRbcX2 and AtRbcX1, respectively, in these studies [18, 19]. The green algae *Chlamydomonas reinhardtii* contains two RbcX-II sequences (CrRbcX-IIa and CrRbcX-IIb, orthologs of AtRbcX-II) and no RbcX-I ortholog. Here we biochemically and structurally characterize CrRbcX-IIa. The crystal structures of CrRbcX-IIa alone and in complex with the C-terminal peptide of RbcL show that CrRbcX-IIa shares the structural topology with cyanobacterial and plant RbcX homologs. However, the RbcL peptide bound to CrRbcX-IIa only occupies part of the central hydrophobic cleft of the RbcX dimer, in contrast to the structure of the cyanobacterial RbcX-peptide complex. Nevertheless, we find that CrRbcX-IIa supports the assembly of cyanobacterial Rubisco, although with reduced efficiency compared to cyanobacterial RbcX-I.

## Materials and Methods

### Plasmids and Proteins

**Fig 1:**
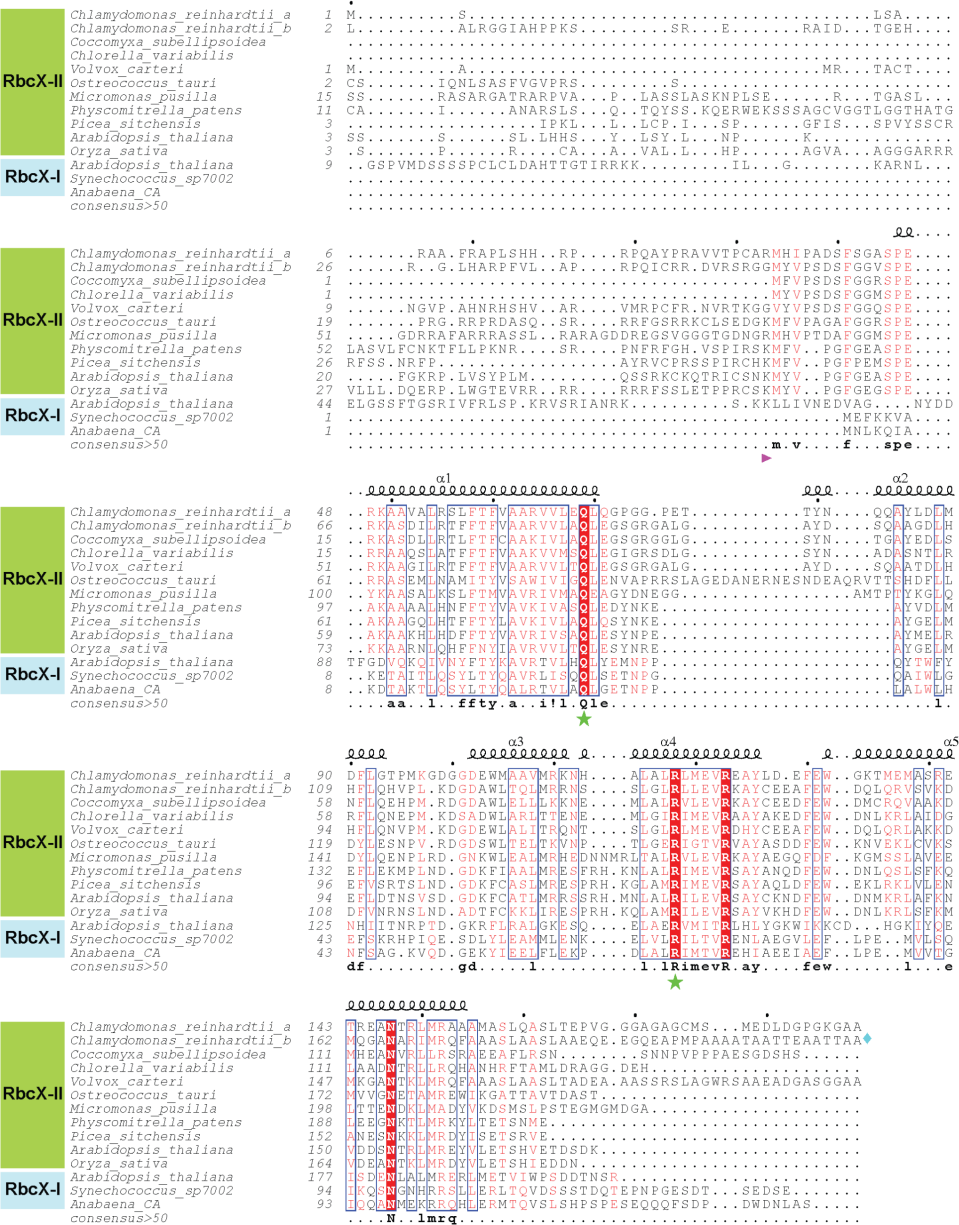
Sequence alignment of RbcX-II from green algae. Amino acid sequences of selected RbcX-II homologs from green algae, mosses and plants were aligned using Clustal-Ω. Note that for the green algae *Coccomyxa subellipsoidea*, *Chlorella variabilis*, *Volvox carteri*, *Ostreococcus tauri* and *Micromonas pusilla* only one RbcX-II sequence is shown. For comparison, RbcX-I from *A*. *thaliana*, *Synechococcus* sp. PCC7002 and *Anabaena sp*. CA are also aligned. All sequence numbering is based on the open reading frames. Secondary structure elements are indicated above the sequences. In the alignment, similar residues are shown in red and identical residues in white using bold lettering on red background. Blue frames indicate homologous regions. The consensus sequence is shown at the bottom. The forward arrow designates the beginning of the mature RbcX-II proteins. The diamond symbol at the end of the CrRbcX-IIb sequence indicates that the sequence continues with 130 amino acids not displayed. Asterisks denote residues known to be essential for RbcX function.

Open reading frames for CrRbcX-IIa were amplified by PCR from *Chlamydomonas reinhardtii* cDNA [20], and cloned between the SacII and SacI restriction sites of the *pHue* plasmid [21], resulting in the following constructs: *pHueCrRbcX-IIa(33–189)*; *pHueCrRbcX-IIa(34–156); pHueCrRbcX-IIa(34–189); pHueCrRbcL(462–474)-RbcX-IIa(37–156)*. The cleavage site for the chloroplast transit peptide of CrRbcX-IIa was predicted based on homology with AtRbcX-II (see Fig 1). The Quik-Change protocol (Stratagene) was used to produce the mutant *pHueCrRbcX-IIa(33–189)(R118A)*. All plasmid inserts were verified by DNA sequencing.

*S*. *elongatus* PCC6301 RbcL_8_S_8_, RbcL_8,_ RbcS, RbcL, GroEL and GroES were purified as previously described [9, 12, 22]. Rabbit antibody against *S*. *elongatus* PCC6301 RbcL was produced using standard procedures.

### Expression and Purification of CrRbcX-IIa

RbcX proteins were expressed as N-terminal His_6_-ubiquitin (His_6_-Ub) fusion proteins in *E*. *coli* BL21 (DE3) cells from *pHue* expression plasmids. Cells were grown to an OD600 of 0.5 at 37°C in LB medium followed by induction for 16 h with 0.5 mM isopropyl-D-thiogalactoside (IPTG) at 23°C. Cells were lysed in 50 mM Tris-HCl pH 8.0, 20 mM NaCl, 1 mM EDTA, 0.5 mg/ml lysozyme and 5 mM phenylmethylsulfonyl fluoride (PMSF) for 30 min on ice, followed by ultrasonication (Misonix Sonicator 3000). The supernatant obtained after high-speed centrifugation (48 000 x g, 40 min, 4°C) was applied to a Ni-IMAC column (GE Biotech) to capture the His_6_-Ub protein, followed by overnight cleavage of the His_6_-Ub moiety at 23°C using the deubiquitinating enzyme Usp2 [21]. All subsequent steps were performed at 4°C. The supernatant was dialyzed against buffer A (20 mM Tris-HCl pH 8.0, 50 mM NaCl) and applied to a pre-equilibrated MonoQ column (GE Biotech). Proteins were eluted with a linear salt gradient from 50 mM to 1 M NaCl. Fractions containing RbcX were combined and concentrated, 5% glycerol was added, followed by flash-freezing in liquid nitrogen and storage at –80°C.

RbcX for crystallographic studies was purified further by Superdex200 (GE Biotech) size exclusion chromatography in buffer A. Protein concentration was determined spectrophotometrically at 280 nm using calculated extinction coefficients.

For selenomethionine (SeMet) labeling by the catabolite repression method [23], the bacteria were grown to mid-log phase at 37°C in M9 medium containing 100 mg L^-1^ ampicillin. Methionine biosynthesis repression was induced by addition of amino acids as follows: 125 mg L^-1^ L-Lys, 100 mg L^-1^ L-Phe, 100 mg L^-1^ L-Tyr, 50 mg L^-1^ L-Ile, 50 mg L^-1^ L-Leu, 50 mg L^-1^ L-Val and 60 mg L^-^1 L-Se-Met. 15 min later the temperature was reduced to 23°C and protein synthesis was induced with 0.5 mM IPTG for 20 h. Cells were harvested and re-suspended in lysis buffer (50 mM Na-phosphate pH 9.0, 300 mM NaCl, 10 mM imidazole and 1 mM β-mercaptoethanol) containing Complete protease (Roche Biotech) inhibitor cocktail. The cells were disrupted by ultrasonication and SeMet-labeled His_6_-Ub RbcX was purified essentially as described above. The protein solution was dialyzed against buffer A containing 1 mM β-mercaptoethanol (β-ME) and applied to a pre-equilibrated MonoQ column. Proteins were eluted with a linear salt gradient from 50 to 400 mM NaCl. Fractions containing SeMet-labeled CrRbcX-IIa(34–156) were subsequently dialyzed against buffer A/β-ME and concentrated. After flash-freezing in liquid N_2_, the protein was stored at -80°C.

### Native Mass Spectrometry (Native-MS)

Purified CrRbcX-IIa(33–189); CrRbcX-IIa(34–156) and CrRbcX-IIa(34–189) (15 μM monomer each) were buffer-exchanged into 100 mM ammonium acetate pH 8.5 (Fluka, Sigma), using Micro Bio-Spin 6 chromatography columns (BioRad). Native-MS analyses were performed in positive ion mode on an electrospray ionization quadrupole time-of-flight (ESI-TOF) hybrid mass spectrometer (Synapt G2-Si, Waters Corp., Manchester, UK) equipped with a Z-spray nano-ESI source. The instrument was mass calibrated using a solution of 30 mg ml^-1^ cesium iodide dissolved in 1:1 acetonitrile:water. Gold-plated 10 μm nano-ESI pipettes (Mascom, Bremen) were used as capillaries. Optimized capillary and sample cone voltages were 1–1.3 kV and 100–150 V, respectively.

### Rubisco Reconstitution

GroEL/ES-mediated RbcL folding was performed as in Liu et al. (2010) with modifications. Denatured *S*. *elongatus* PCC6301 RbcL was diluted 200-fold from 6 M GuHCl (final RbcL concentration 0.5 μM) into ice-cold buffer B (20 mM MOPS-KOH pH 7.5, 100 mM KCl, 5 mM MgOAc_2_) containing GroEL (1 μM oligomer), 1 mg/ml BSA and 5 mM DTT. The reaction was incubated on ice for 60 min, followed by centrifugation to remove any aggregated RbcL. GroES (2 μM oligomer), RbcX (2 μM AnaCa-RbcX or 30 μM CrRbcX dimer) and *S*. *elongatus* PCC6301 RbcS (5 μM) were added to the supernatant containing GroEL bound Syn6301-RbcL as indicated in Figure legend. Reconstitution was initiated by addition of 4 mM ATP at 25°C. Reactions were stopped by addition of apyrase (Sigma) to a final concentration of 0.25 U/μl to inhibit GroEL/ES activity.

For measurement of Rubisco enzymatic activity at 25°C, the reaction was supplemented with Syn6301-RbcS (5 μM) and C-terminal RbcL peptide (KEIKFEFETMD) of *S*. *elongatus* PCC6301 (200 μM) when indicated, and assembly of RbcL_8_S_8_ allowed to proceed for 15 min before enzymatic assay. Rubisco carboxylation activity was determined after incubation for 10 min in 50 mM Tris-HCl pH 8.0, 10 mM MgCl_2_, 30 mM NaH^14^CO_3_ (25 Bq/nmol) and the amount of carbon fixed was quantified [24]. Activities are expressed as percent of purified Syn6301-RbcL_8_ (~0.06 μM oligomer) standard supplemented with RbcS (5 μM).

### Crystallization

Crystals of CrRbcX-IIa(34–156) were grown using the hanging drop vapor diffusion method at 20°C by mixing 1 μl protein sample at 6 g L^-1^ and 1 μl reservoir solution. Crystals of SeMet-labeled CrRbcX-IIa(34–156) resembling shields were obtained with a reservoir solution containing 5% PEG-3350/0.2 M MgCl_2_/50 mM Tris-HCl pH 8.0. For cryo-protection, the crystals were transferred into mother liquor containing 15% PEG-3350/0.2 M MgCl_2_/50 mM Tris-HCl pH 8.0, followed by stepwise increase to 20% glycerol content and flash-freezing in liquid nitrogen.

Crystals of the CrRbcL(462–473)-RbcX-IIa(37–156) fusion protein were grown by the hanging drop vapor diffusion method at 20°C using 0.1M Tris-HCl pH 8.5, 25% PEG2000 monomethyl ether as precipitant.

### Structure Solution and Refinement

The diffraction data were collected at beamline X10SA of the Swiss Light Source (SLS) in Villigen, Switzerland. Diffraction data were integrated and scaled with XDS [25]. Pointless [26], Scala [27] and Truncate [28] were used to convert the data to CCP4 format, as implemented in the CCP4i interface [29].

The structure of CrRbcX-IIa(34–156) was solved by Se-SAD using crystals from SeMet-labeled protein at 2.0 Å resolution. 36 selenium sites were found by direct methods using SHELXD as implemented in HKL2MAP [30, 31]. SHELXE was used for density modification and auto-building of a poly-alanine model. The resulting map was readily interpretable and the sequence was docked using Coot [32]. The final model was created by iterative Coot model building and Refmac5 refinement cycles [33]. The structure of the fusion protein CrRbcL(462–474)-RbcX-IIa(37–156) was solved by molecular replacement using Molrep [34], and the models modified and refined as above. Residues facing solvent channels with disordered side chains were modeled as alanines. Coordinates were aligned with Lsqkab and Lsqman [35]. Figures were generated with the program PyMOL [36] and ESPript [37]. Coordinates and structure factor amplitudes were deposited to Protein Data Bank under accession codes 5BS1 and 5BS2.

## Results

### Structural Analysis of *Chlamydononas reinhardtii* RbcX

**Fig 2:**
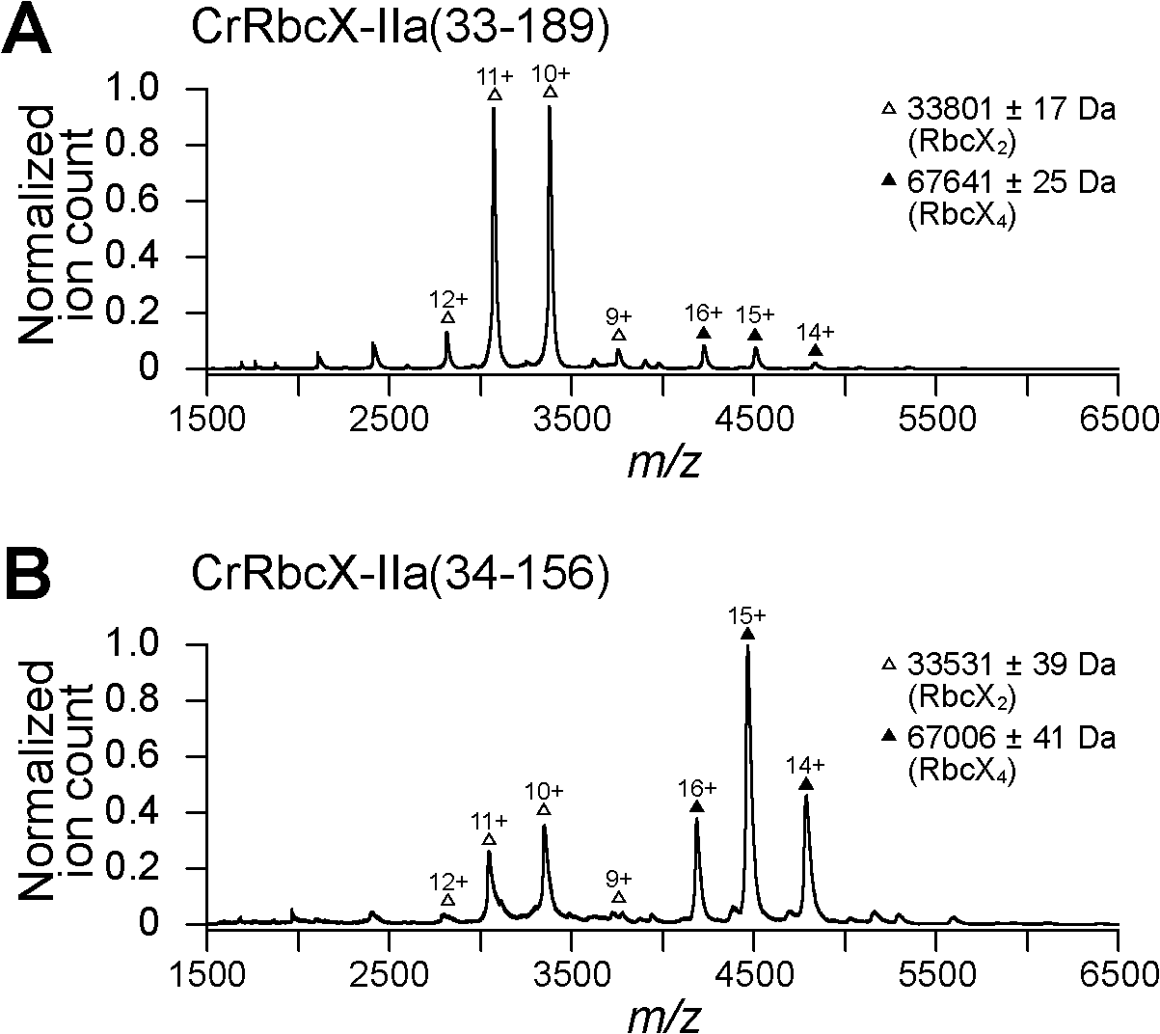
Oligomeric state of CrRbcX-IIa analyzed by native-MS. Nano-ESI native-MS spectra of CrRbcX-IIa(33–189) (A) and CrRbcX-IIa(34–156) (B). Symbols indicate the charge state distributions with the charge states shown for some peaks; the calculated mass around the *m/z* values of the respective protein complexes is reported. The accuracy of mass values calculated from the different *m/z* peaks is indicated.

The genome of *C*. *reinhardtii* contains no RbcX-I, but instead has two RbcX-II genes, *g688*.*t1* (locus Cre01.g030350) and *g7885*.t1 (locus Cre07.g339000). We refer to the gene products as CrRbcX-IIa and CrRbcX-IIb, respectively. Note that in the most recent genome annotation CrRbcX-IIa would start at amino-acid residue 34 and lacks the sequence encoding the transit peptide. CrRbcX-IIb, on the other hand, has a putative transit peptide but the annotated gene codes for a protein twice the length of other RbcX homologs (~290 residues) with only the first ~160 amino acids displaying homology to RbcX proteins (Fig 1). The additional sequence in CrRbcX-IIb probably represents an intron, and thus the sequence for CrRbcX-IIb is apparently incorrectly annotated. We focused our analysis on CrRbcX-IIa, which was previously annotated with a putative transit peptide. Based on sequence alignment with the mature form of *A*. *thaliana* RbcX-II (also known as AtRbcX1), which begins with Lys46 [19], we cloned CrRbcX-IIa starting at Arg33 (Fig 1), generating a protein of ~17 kDa. CrRbcX-IIa(33–189) was recombinantly expressed and purified from the soluble fraction. Analysis by native-MS showed that CrRbcX-IIa is a dimer in solution, as expected (Fig 2A).

**Fig 3:**
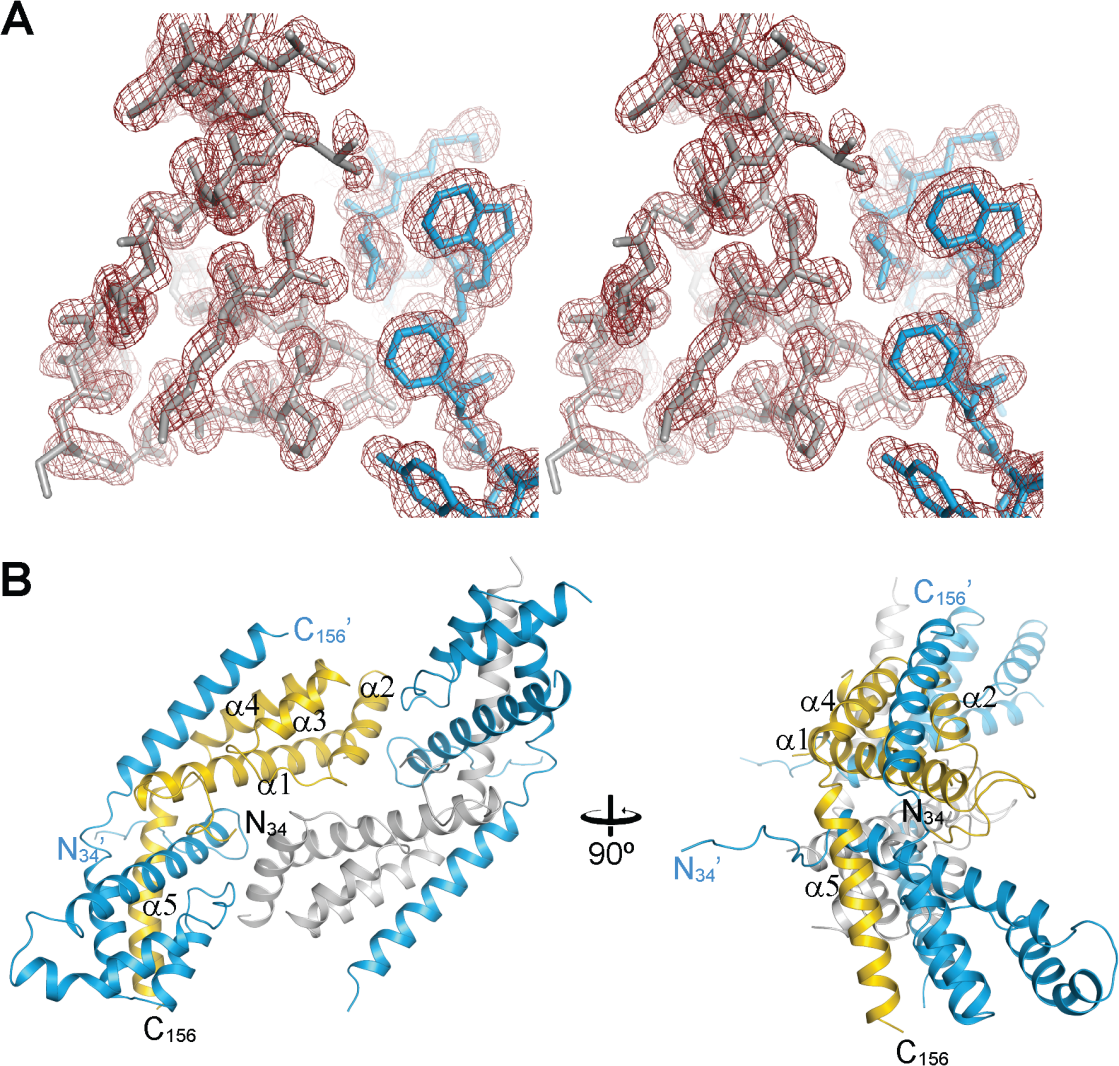
Asymmetric unit of the CrRbcX-IIa(34–156) crystal. (A) Stereoview of a representative portion of the experimental density map at 1.0 σ. The final model is superposed in stick representation. (B) Tetrameric complex of the SeMet-labeled oligomer in the asymmetric unit of the crystal lattice. Two perpendicular views are shown. On the left, a view along the two-fold molecular axis is shown. CrRbcX-IIa(34–156) is shown in ribbon representation. In each dimer, one of the chains is colored blue and the other gold or silver, respectively. Chain termini and secondary structure elements are indicated. The N-termini of the golden/silver subunits reach into the clefts (roughly horizontal); the N-termini of the blue subunits towards crystallographic symmetry mates.

**Fig 4:**
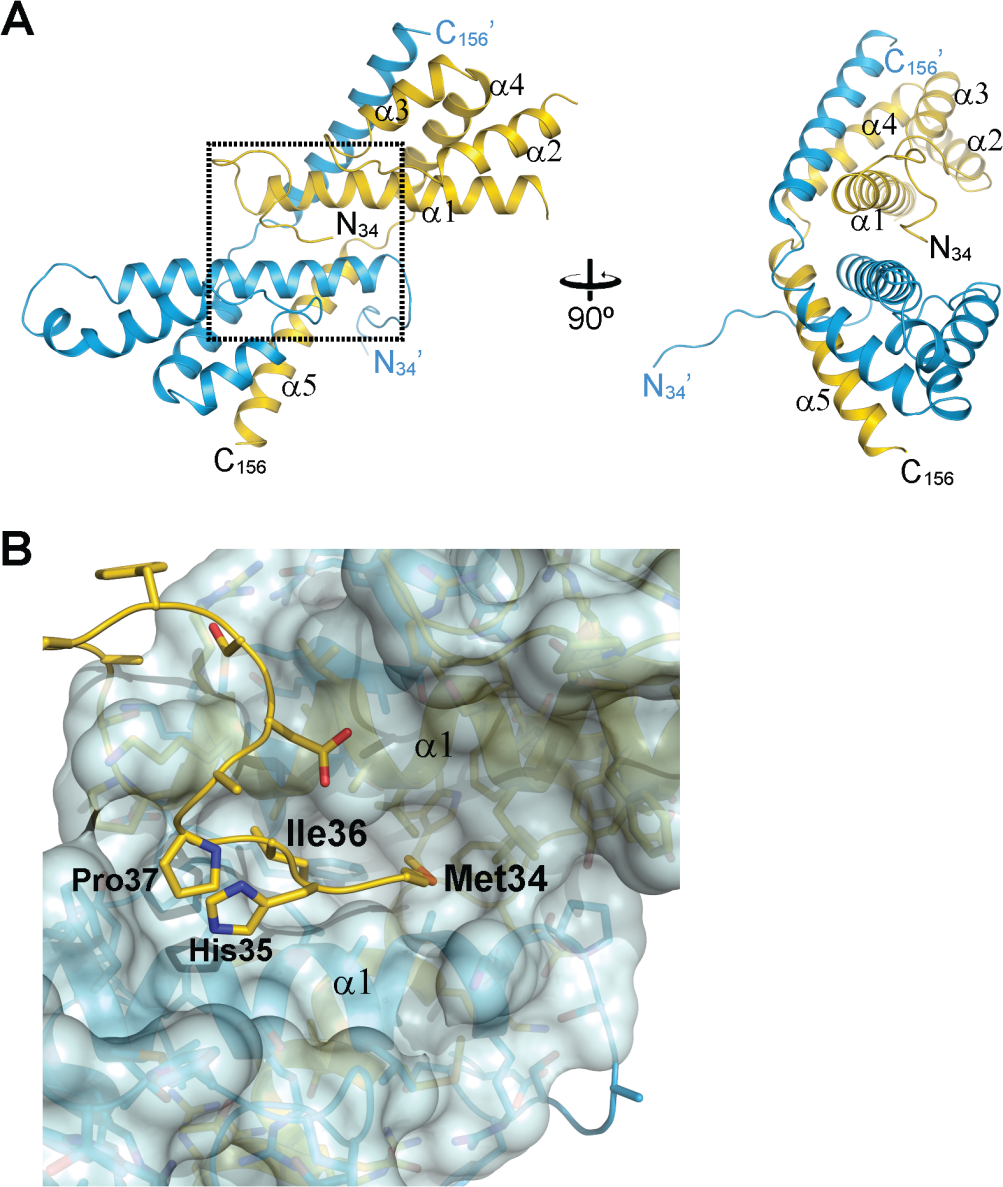
Crystal structure of the CrRbcX-IIa(34–156) dimer. (A) Ribbon representation of the CrRbcX-IIa(34–156) dimer. Two perpendicular views are shown, the first along the molecular two-fold axis. (B) Interactions of the N-terminal tail with the hydrophobic cleft in CrRbcX-IIa(34–156). A zoom-in on the boxed area in panel (A) is shown. The N-terminal tail is shown as a coil with prominent sidechains in stick representation. The bulk of the CrRbcX-IIa(34–156) is represented as a molecular surface.

Full-length CrRbcX-IIa failed to crystallize. A stable fragment comprising residues 34–156 lacking the flexible C-terminal 33 residues was produced by subtilisin treatment, as determined by mass spectrometry (MS). An unstructured C-terminus was also found to be present in the cyanobacterial Syn7002-RbcX and was not required for function in Rubisco assembly [12]. We recombinantly expressed and purified the truncated CrRbcX-IIa(34–156) protein for further structural analysis. The structure of the selenomethionine (SeMet)-labeled CrRbcX-IIa(34–156) protein was solved by selenium-single-wavelength anomalous dispersion (Se-SAD) at 2.0 Å resolution. The experimental electron density was readily interpretable (Fig 3A). The structural model was built against data to 1.6 Å resolution and refined to final R and R_free_ values of 0.177 and 0.206, respectively (see Table 1 for data collection and refinement statistics). The asymmetric unit of the monoclinic unit cell contains four copies of CrRbcX-IIa(34–156) in a two-fold symmetric topology (Fig 3B). Each chain consists of a succession of five α-helices. In three of the subunits the insertion after helix α1, residues 73–77, is disordered. This insertion is typical for RbcX-II sequences from green algae (Fig 1). Apart from the N-terminal 10 residues (see below), the backbones of the CrRbcX-IIa(34–156) subunits are closely similar (r.m.s.d. of Cα positions of 0.267 to 0.577 Å). The subunits form arch-shaped, two-fold symmetric dimers with a hydrophobic cleft in the center (Fig 4A), similar to other known RbcX structures [12, 17, 18]. In each subunit helices α1-α4 form a four-helix bundle, which associates with helix α5 of the opposing subunit in the dimer (Fig 4A). The N-terminal sequence of one subunit binds into the central cleft, with residues Met34 and Ile36 reaching into hydrophobic pockets located between the anti-parallel helices α1 and α1’ at the bottom of the cleft (Fig 4B). The N-terminal ammonium group of Met34 engages in a tight salt bridge (lengths 2.53 and 2.58 Å) with Asp90 from the opposing dimer, which presumably stabilizes the tetramer arrangement. The other N-terminal peptide inserts into a cleft between neighboring tetramers in the crystal lattice. The dimers in the asymmetric unit interact substantially (1370 Å^2^ accessible surface area buried on each dimer). Indeed, CrRbcX-IIa(34–156) formed mainly tetramers in solution as detected by native-MS (Fig 2B). However, this interaction is unlikely to be functionally relevant since full-length CrRbcX-IIa behaved as a dimer in solution (Fig 2A).

**Table 1:** Crystallographic data collection and model refinement statistics.

Dataset	CrRbcX-IIa(34–156) (SeMet)	CrRbcL(462–474)-CrRbcX-IIa(37–156)
**Data collection**		
Wavelength (Å)	0.9790	0.9999
Space group	*P*1	*P*1
Cell dimensions		
a, b, c (Å);	36.13, 52.99, 61.56;	34.22, 38.53, 50.36;
α, β, γ (°)	76.49, 81.10, 70.10	88.47, 81.53, 67.92
Resolution limits (Å)*	59.66–1.6 (1.69–1.6)	35.68–1.97 (2.07–1.97)
R_merge_ *	0.059 (0.332)	0.081 (0.494)
I/sigma *	18.3 (4.6)	10.4 (2.0)
Multiplicity *	7.1 (6.7)	2.4 (2.2)
Completeness (%) *	94.1 (77.2)	94.2 (87.9)
Wilson B-factor (Å^2^)	15.3	22.8
**Refinement**		
Resolution range	30–1.6	30–1.97
Reflections **	49112 (2585)	15009 (1887)
*R*_*work*_ / *R*_*free*_	0.177 / 0.206	0.200 / 0.222
Number of atoms	4185	1887
Average B-factor (Å^2^)	19.0	31.0
r.m.s. deviations		
Bond length (Å)	0.011	0.013
Bond angle (°)	1.509	1.423
Ramachandran plot ***		
Favoured (%)	97.7	97.3
Allowed (%)	2.1	2.2
Outliers (%)	0.2	0.4

* Values in parenthesis for outer shell.

** Values in parenthesis for test set.

*** Values from Molprobity 4.02.

### Comparison with Other RbcX Structures

**Fig 5:**
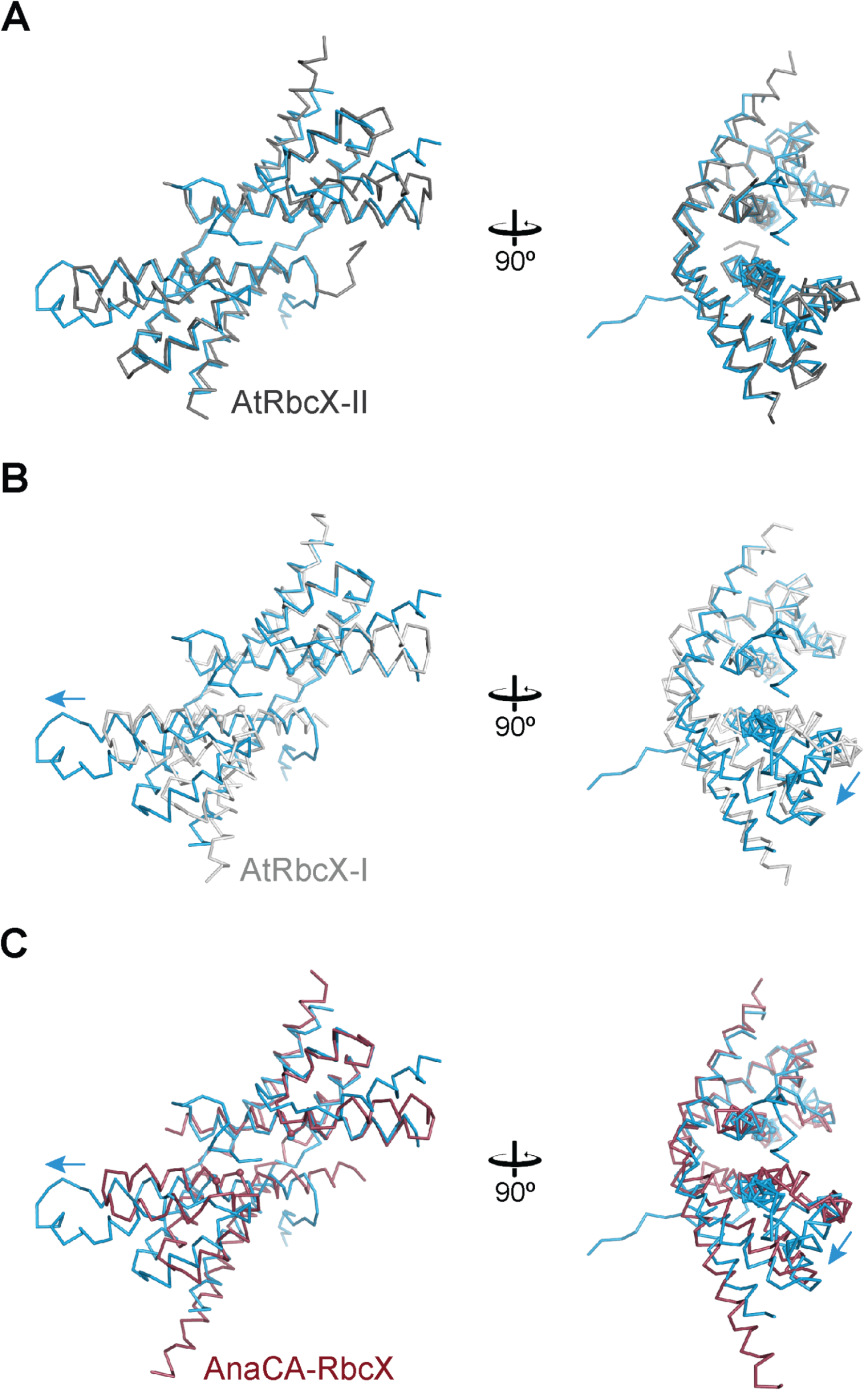
Comparison of the CrRbcX-IIa(34–156) structure with RbcX-II and RbcX-I homologs. (A) Comparison with the *A*. *thaliana* homolog AtRbcX-II. The backbones of the subunits of CrRbcX-IIa(34–156) are represented as a Cα trace in the same views as in Fig 4A. Spheres designate the Cα atoms of Leu57 and Phe60 in CrRbcX-IIa, or the respective sequence positions in the homologous proteins. CrRbcX-IIa and AtRbcX-II are shown in blue and dark grey, respectively. (B) Comparison with the *A*. *thaliana* homolog AtRbcX-I, which is shown in light grey. (C) Comparison with cyanobacterial AnaCA-RbcX which is shown in red. Arrows indicate the direction of displacement of the second 4-helix bundle of CrRbcX-IIa(34–156).

The crystal structure of the dimer of CrRbcX-IIa(34–156) is closely similar to that of the plant ortholog AtRbcX-II (AtRbcX1) [18] (Fig 5A). 175 Cα positions could be superposed with a r.m.s.d. of 1.239 Å. In contrast, the structure of CrRbcX-IIa(34–156) differs more substantially from the structures of cyanobacterial RbcX and AtRbcX-I. For example, while one four-helix bundle and the associated C-terminal helix from the other subunit of the dimer of AtRbcX-I are reasonably well superimposable with CrRbcX-IIa(34–156) (r.m.s.d. 1.414 Å for 120 matching Cα atom positions), the other helical bundle is markedly shifted (Fig 5B). The situation is closely similar when comparing with the cyanobacterial *Anabaena sp*. CA RbcX (AnaCA-RbcX) with an r.m.s.d. 1.453 Å for 134 matching Cα atom positions (Fig 5C). The rearrangement displaces helices α1 and α1’ in the protomers longitudinally, which moves the symmetry-related pairs of hydrophobic pockets apart by ~5 Å. This becomes apparent from comparing the positions of residues Leu57 and Phe62, which line the hydrophobic pockets (spheres in Fig 5). Consequently, a pseudo-symmetrical binding of the FEF motif in the RbcL C-terminal peptide across the dyad axis is not possible in CrRbcX, in contrast to the binding mode of the FEF motif to cyanobacterial RbcX [10, 12]. The helices α2 of CrRbcX-IIa(34–156), which form the “walls” of the hydrophobic cleft, are rotated outwards in comparison to cyanobacterial RbcX (Fig 5C), widening the cleft.

### Structural Basis of RbcL Peptide Recognition

**Fig 6:**
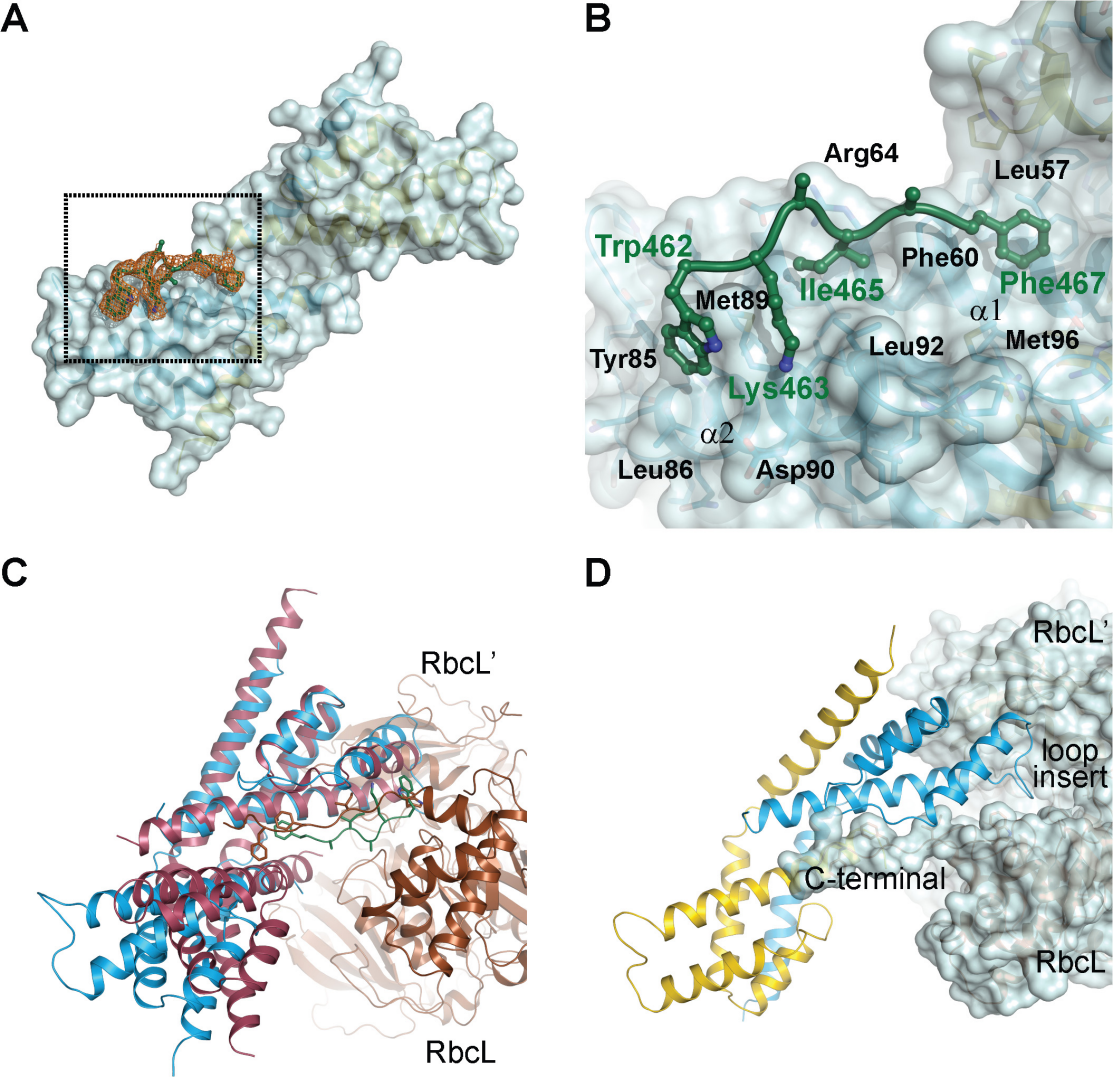
Crystal structure of a fusion protein revealing the interactions between CrRbcX-IIa and the C-terminal tail of CrRbcL. (A) Unbiased omit difference electron density for the RbcL tail residues of the CrRbcL(462–474)-RbcX-IIa(37–156) fusion protein. The C-terminal sequence of CrRbcL is shown as a coil and the sidechains in stick representation. The difference electron density at 1.5 σ level is shown as orange meshwork. CrRbcX-IIa(37–156) is represented as a molecular surface. (B) Detailed view of the RbcL-RbcX interactions. The area boxed in panel (A) is shown. (C) Superposition of the CrRbcX-IIa(37–156) onto the Syn6301-RbcL_8_/AnaCA-RbcX_8_ crystal structure [10]. The structures are shown in ribbon representation. The RbcL subunits are shown in brown and siena; the AnaCA-RbcX dimer in red; CrRbcX-IIa dimer in blue. (D) Putative contacts of CrRbcX-IIa(37–156) with the surface of the Syn6301-RbcL_8_ complex. The same view as in panel (C) is shown.

Attempts to obtain a co-crystal between CrRbcXIIa(34–159) and the C-terminal RbcL peptide failed, presumably due to low peptide binding affinity. Taking advantage of the finding that the N-terminus of RbcX binds into the central cleft (Fig 4), we therefore generated a fusion construct between CrRbcX-IIa and the C-terminal recognition motif in CrRbcL. In this construct, residues 462–473 of CrRbcL (sequence WKEIKFEFDTID) are directly linked to residue Pro37 at the N-terminus of CrRbcX-IIa(37–156), with the new N-terminus of the fusion protein starting with Trp462 of the RbcL sequence. This fusion protein readily crystallized and the structure was solved at 1.97 Å resolution (Table 1). The structural core of CrRbcX-IIa(37–156) in the fusion protein is virtually identical to that obtained for CrRbcX-IIa(34–156) (r.m.s.d. 0.425 Å for 211 matching Cα positions). Thus it is unlikely that the contact area with the RbcL peptide is distorted by crystal packing. Difference electron density along the hydrophobic cleft could be assigned to the RbcL residues 462–467 (WKEIKF). Residues 468–473 (EFDTID) of RbcL as well as residues 37–43 of CrRbcX-IIa were disordered (Fig 6A). Notably, Phe469 was among the disordered residues, consistent with the finding that the corresponding Phe464 in Syn7002-RbcL is functionally less important for RbcX binding than Phe462 (Phe467 in CrRbcL) [12]. The sidechains of Ile465 and Phe467 point into hydrophobic pockets surrounded by Phe60/Arg64/Leu67/Leu92 and Leu57/Phe60/Met96, respectively (Fig 6B). The sidechain of Lys463 points towards the C-terminal end of helix α2 and Asp90. The indole moiety of Trp462 interacts with Tyr85 and Met89, but also with a neighboring CrRbcX-IIa molecule (not shown), and thus these interactions seem to be influenced by crystal packing.

Superposition with the structure of the heterologous cyanobacterial Syn6301-RbcL_8_/AnaCA-RbcX_8_ assembly intermediate [10] shows that Ile465 and Phe467 of CrRbcL are recognized by similar sites on CrRbcX-IIa (Fig 6C). The peptide is oriented more towards helix α2 in the cyanobacterial structure, whereas it assumes a deeper and more central position in the hydrophobic cleft of CrRbcX-IIa (Fig 6C). The indole ring of Trp462 is at roughly the same place in the superposition, but the backbone conformations differ strongly at this segment. We note that in the context of the RbcL subunit this residue would be connected, whereas it forms the N-terminal residue in the fusion construct. This difference in sequence topology may influence the binding mode.

The superposed CrRbcX-IIa is compatible with the surface of the RbcL anti-parallel dimer in the context of the RbcL_8_ core complex (Fig 6D), in a topology similar to that observed for the cyanobacterial RbcX [10]. The C-terminal sequence of one RbcL subunit reaches into the central cleft of CrRbcX-IIa and the functionally critical, conserved residues Gln69 and Arg118 (Fig 1) are positioned correctly for interaction with the second RbcL subunit (Fig 6D). The loop insertion between helices α1 and α2 of CrRbcX-IIa, which is ordered in the structure of the CrRbcL(462–474)-RbcX-IIa(37–156) fusion protein, would extend into a shallow groove of the RbcL dimer surface (Fig 6D). We speculate that this loop insertion found in RbcX sequences of green algae might modulate the interaction with RbcL.

### Functional Characterization of CrRbcX

**Fig 7:**
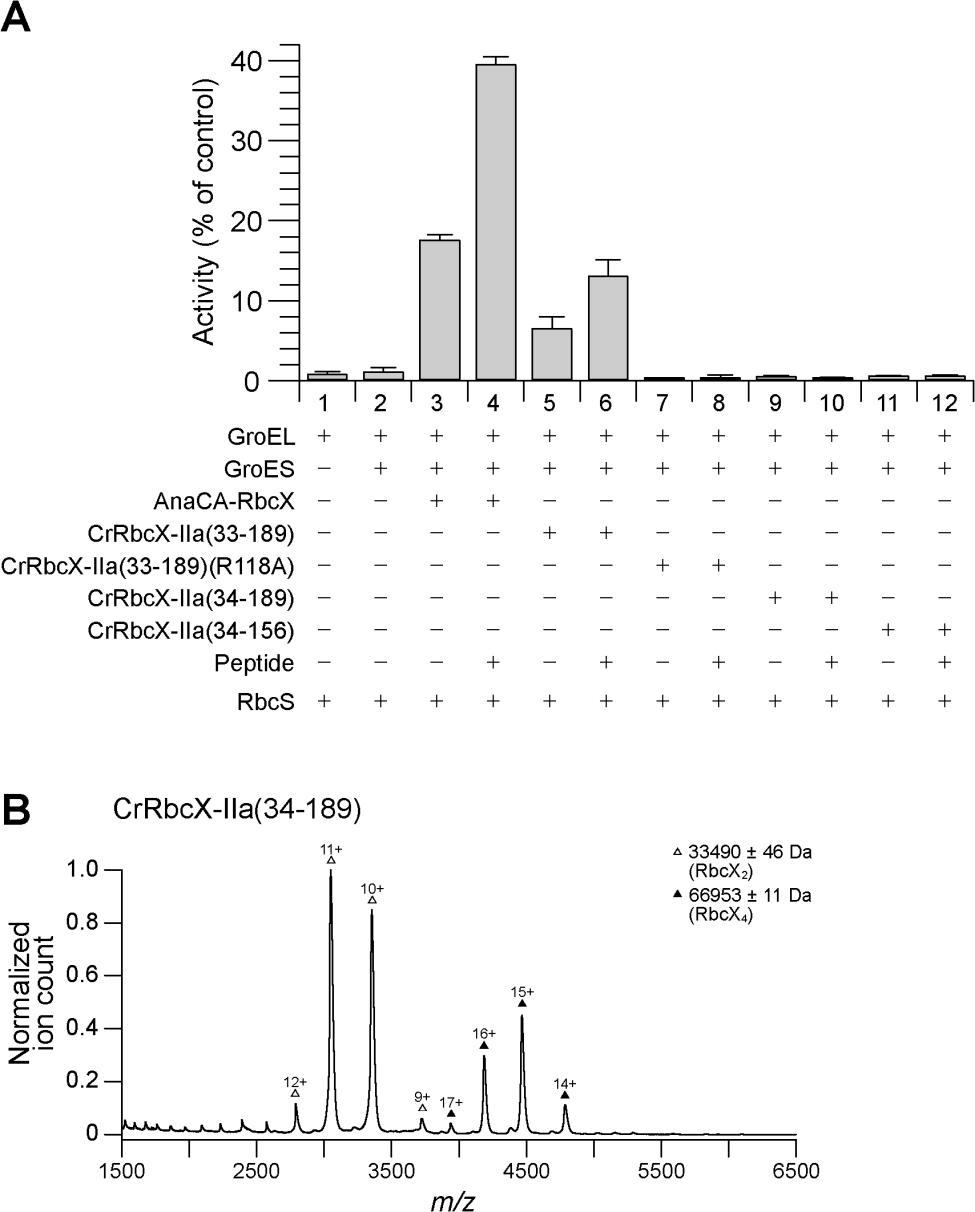
Rubisco reconstitution of CrRbcX-IIa and oligomeric state of CrRbcX-IIa(34–189) analyzed by native-MS. (A) Rubisco reconstitution. Chemically denatured RbcL from *S*. *elongatus* PCC6301 (at 100 μM) was diluted 200-fold into ice-cold buffer containing GroEL (1.0 μM). The components (2 μM GroES oligomer; 2 μM AnaCa-RbcX or 30 μM CrRbcX dimer) were added as indicated and refolding/assembly initiated by addition of 4 mM ATP at 25°C (see Materials and Methods). After incubation for 60 min, RbcS (5 μM) was added with or without C-terminal RbcL peptide (200 μM) for 15 min, followed by Rubisco enzyme assay. The activity of RbcL_8_ core complex (~0.06 μM oligomer) incubated with RbcS (5 μM) was set to 100%. Error bars s.d. (n = 3 independent experiments). (B) Nano-ESI native-MS spectra of CrRbcX-IIa(34–189). Symbols indicate the charge state distributions with the charge states shown for some peaks; the calculated mass around the *m/z* values of the respective protein complexes is reported. The accuracy of mass values calculated from the different *m/z* peaks is indicated.

We used the previously reconstituted Rubisco from *S*. *elongatus* PCC6301 [9] and the bacterial chaperonin system GroEL/ES to assess the functionality of CrRbcX-IIa in Rubisco assembly. Unfolded RbcL was bound to GroEL upon dilution from denaturant. Assembly was initiated by adding GroES, ATP and RbcX for 60 min at 25°C, followed by addition of RbcS for Rubisco activity assay. The formation of holoenzyme was dependent on RbcX as shown previously [9], reaching a yield of ~20% with AnaCA-RbcX (Fig 7A). Addition of the C-terminal RbcL peptide prior to RbcS doubled the yield to ~40% by facilitating the displacement of RbcX from the RbcL_8_RbcX_8_ assembly intermediate by RbcS [9, 10]. A lower yield of enzyme activity of ~7% was obtained with full-length CrRbcX-IIa(33–189) protein, but only when present at a high molar excess (30 μM dimer) over RbcL. Again the activity doubled in the presence of the C-terminal RbcL peptide (Fig 7A). The mutant CrRbcX-IIa(R118A) did not support assembly, consistent with this conserved residue being involved in the stabilization of the RbcL dimer [9, 10, 12]. Notably, CrRbcX-IIa(34–189), lacking the N-terminal residue Arg33 of the full-length protein was inactive (Fig 7A). Arg or Lys is conserved at this position among most RbcX-II homologs (Fig 1). Arg33 is also missing in the C-terminally truncated, crystallized CrRbcX-IIa(34–156) protein. In the crystal structure, the amino group of the N-terminal Met34 forms a short salt bridge (2.53–2.58 Å) with Asp90 from the other RbcX dimer, which appears to stabilize the tetramer. In addition, Arg33 would clash with the other dimer, consistent with the MS data showing that deletion of Arg33 favors tetramer formation in solution (Fig 2). We suggest that in the absence of Arg33, the N-terminus of RbcX may bind into the central cleft, rendering the protein non-functional in Rubisco assembly (Fig 7A), consistent with the formation of non-functional tetramers (Fig 7B).

## Discussion

Our data demonstrate that RbcX-II from the green algae *C*. *reinhardtii* functions as a bona fide Rubisco assembly chaperone, despite its considerable evolutionary distance from cyanobacterial and eukaryotic RbcX-I proteins. Like all other known RbcX proteins, CrRbcX-IIa is an arc-shaped dimer with a central hydrophobic cleft that binds the C-terminal sequence of the RbcL subunit. Conserved polar residues at the corners of RbcX make critical contacts to the N-domain of an adjacent RbcL, thereby stabilizing the RbcL anti-parallel dimer in a state competent for assembly to the RbcL_8_ core complex of Rubisco.

The crystal structure of CrRbcX-IIa differs from the structures of cyanobacterial RbcX homologs in several aspects. The adjacent helices α1, which form the floor of the hydrophobic cleft, are shifted with respect to each other, moving the binding pockets for the two Phe side-chains in the C-terminal RbcL binding motif apart. Consistently, density for the bound peptide sequence is only discernible until the first Phe residue (Phe467) in the complex structure. There are additional hydrophobic cavities between the helices close to the symmetry axis, resulting from the conserved substitution of Thr10 in cyanobacterial RbcX by Ala in RbcX-II (residue 50 in CrRbcX-IIa sequence numbering), but these volumes are not occupied in the complex with peptide. In the apo-structure, the sidechains of the conserved residues Met34 and Ile36 point into these pockets, but the functional significance of this interaction, if any, is unclear. Interestingly, in the structure of the *A*. *thaliana* ortholog, which has essentially the same backbone conformation, the pockets are smaller and intra-molecular binding of the N-terminus into the central cleft is not observed.

Besides the RbcX homologs, a recent screen of a Maize mutant library identified several additional Rubisco accumulation factors, including Bsd2, Raf1 and Raf2 [38–42]. RbcX and Raf1 are generally conserved in photosynthetic organisms containing form IB Rubisco [2, 3], but mediate assembly by different mechanisms [43]. Whether RbcX and Raf1 cooperate in a coherent assembly pathway or act in parallel pathways is still unknown.
